# Measurement of Longitudinal Chromatic Aberration in the Last Crystalline Lens Surface Using Hartmann Test and Purkinje Images

**DOI:** 10.3390/s22072653

**Published:** 2022-03-30

**Authors:** Uriel Calderon-Uribe, Geovanni Hernandez-Gomez, Armando Gomez-Vieyra

**Affiliations:** 1 Multidisciplinary Studies Department, Engineering Division, Campus Irapuato-Salamanca, University of Guanajuato, Guanajuato 38944, Mexico; u.calderonuribe@ugto.mx; 2 Laboratorio de Sistemas Complejos, Departamento de Ciencias Básicas, Universidad Autónoma Metropolitana, Unidad Azcapotzalco, Av. San Pablo 180, Ciudad de México 02200, Mexico; agvtex@gmail.com

**Keywords:** Hartmann test, chromatic aberration, Zernike polynomials, longitudinal chromatic aberrations, Purkinje images, Purkinje image detection

## Abstract

Research has shown that longitudinal chromatic aberration (LCA) of the human eye is generated across all of the eye’s optical surfaces. However, it may not be necessary to measure the LCA from the first surface of the cornea to the retina, as it is known that most of the changes that can modify the path of light occur from the first surface of the cornea to the last surface of the crystalline lens. This investigation presents the study of an objective technique that allows the measurement of longitudinal chromatic aberration (LCA) on the last crystalline lens surface by developing a pulse width wavefront system using a Hartmann test, Purkinje image, and Zernike polynomial. A blue pulse (440–480 nm) and a red pulse (580–640 nm) were used to generate a pattern of spots in the human eye. This pattern generated on the posterior surface of the crystalline lens of the human eye allows the reconstruction of the wavefront via a modal method with Zernike polynomials. Once the wavefront is reconstructed, Zernike coefficients can be used to quantify the LCA. The methodology and objective measurements of the magnitude of the longitudinal chromatic aberration of five test subjects are explained in this article.

## 1. Introduction

Due to the presence of aberrations in the human eye (monochromatic aberrations and chromatic aberrations), the quality of vision is affected. Currently, aberrations are measured using wavefront sensors with infrared (monochromatic) light. However, since the human eye perceives chromatic light, studies that measure the quality of vision should consider the aberration that is generated in the visible spectrum, and its effects on visual quality.

Chromatic aberration in the eye arises from the dependence of the wavelength on the refractive index of the ocular medium, generating chromatic dispersion [1,2,3]. Ocular color dispersion includes three major optical effects: the color difference in focus, magnification, and position. The former relates to longitudinal chromatic aberration (LCA), whereas the latter two are known as transverse chromatic aberration (TCA) [4,5]. LCA has been described in several reports that include subjective methods [6,7,8] and objective methods [9,10,11]. It is well known that longitudinal chromatic aberration in an adult is approximately 2D across the visible spectrum (400 to 700 nm) with little variability among people [12,13]. By comparison, TCA is commonly measured through subjective methods, using two wavelengths and Vernier alignment [1,14,15,16]. However, there are also image-based methods using an adaptive optics scanning laser ophthalmoscope (AOSLO) [17,18,19]. Since LCA varies little between individuals, a fixed correction between the relative source of vergence for multi-wavelength imaging and simulation is sufficient. Nonetheless, TCA is more difficult to assess but can be reliably measured with an image-based method using AOSLO [1]. This measurement is made by obtaining the position of the pupil related to the incoming beam [18] and, consequently, the measurement will change dynamically with small variations in the position of the pupil.

TCA knowledge has become important for image quality estimation [5,8], since it has a great impact on chromatic monocular diplopia and chromostereopsis [16,20]. Furthermore, both chromatic aberration and monochromatic aberration interact with each other, which leads to the presence of monochromatic aberration to mitigate the impact of the chromatic aberration [21,22]. Thus, attempts to correct LCA and TCA have shown benefit in improving the quality of vision [23,24,25].

Due to the interaction of both chromatic and monochromatic aberrations (generated under polychromatic light), the quality of vision is affected. A significant amount of research suggests that monochromatic aberration plays an important role in the generation of chromatic aberration [21,26,27], which can explain why some optical devices are incapable of improving the quality of vision unless both aberrations are corrected [28]. However, for the correction of chromatic aberrations, devices that quantify the deformation must first be developed.

Different subjective techniques across different wavelengths have been used to measure LCA [4,12]. The results show that for a spectral range of 365–750 nm, an LCA of 3.2 D is obtained [12], and for a range of 450–650 nm, an LCA value of 1.33 D is obtained [8]. The information obtained from the subjective tests is used to find the Cornu expression for the dependence of the refractive index with a wavelength in the 400–700 nm range.

In addition to subjective techniques, objective measurements, such as reflectometric techniques (double-pass retinal images) [9] or using a point source of light at different wavelengths [11], has been used to measurement the LCA. Research on reflectometry shows that the LCA in ranges of 460–700 nm is approximately 1.4 D [9] and in ranges of 458–632 nm is approximately 1.0 D [11]. Objective measurements have allowed us to measure the chromatic difference in focus between two wavelengths (532–787 nm), obtaining a value of 0.72 D [10], and in the near infrared (700–900 nm), a value of 0.4 D. [26,29]. New reflectometry techniques have made it possible to design new devices that improve visual quality in the human eye (intra-ocular lenses).

Despite the differences in the techniques mentioned above, there is a similarity between these techniques, which all measure the total aberration of the eye (from the first surface of the cornea to the retina). However, it may not be necessary to measure the chromatic aberration of the entire eye. It is known from the physiology of the human eye [30] that, from the first surface of the cornea to the last surface of the crystalline lens, different refractive indices alter the direction of light, compared to the last surface of the eye, which is only composed of the vitreous humor (there is only one refractive index). This assumption makes us think that the highest concentration of chromatic aberration of the human eye can be generated from the first surface of the cornea to the last surface of the crystalline lens (due to changes in the refractive index), and the vitreous humor only contributes a small amount to chromatic aberration. Due to the lack of techniques currently available to verify this theory, the presented methodology was proposed.

In this paper, a method based on the calculation of an objective measure of LCA is described. Aberration was measured using a blue pulse (440–460 nm) and a red pulse (580–640 nm). These pulse widths are used to generate a pattern of spots on the last surface of the crystalline lens. The main objective of this work was to measure the longitudinal chromatic aberration in the last surface of the lens and to compare it with previous research. Subsequently, and using the Zernike polynomials, the LCA is calculated. These pulse widths were chosen as they approximate what a human being can see without harming a person’s sight. The remainder of this paper is structured as follows: in Section 2, the principles used for the measurement of the wavefront are described. In Section 3, the development and implementation of the built device are presented. Section 4 describes the image segmentation process. The chromatic aberration measurement method is introduced in Section 5. Measurements and results are described in Section 6. Finally, the conclusion of this study is present in Section 7.

## 2. Principles and Methods Used to Measure the Longitudinal Chromatic Aberration

### 2.1. Purkinje Images

**Figure 1:**
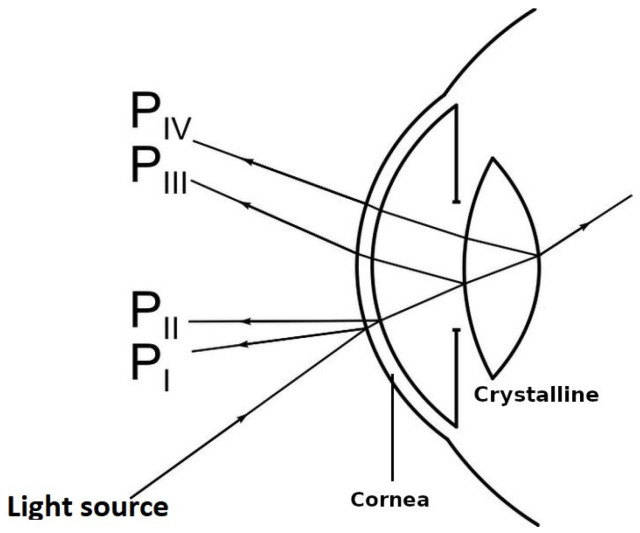
Purkinje images generated on the optical surfaces of the eye.

There are two optical elements in the human eye that allow light to be focused on the retina: the cornea (anterior and posterior) and the crystalline lens (anterior and posterior). The light that enters the eye forms an image on the anterior surface of the cornea, known as the first Purkinje image (PI), shown in Figure 1. This light also forms a second Purkinje image (PII) on the posterior surface of the cornea, which mostly coincides with the PI image.

The light that is not reflected by the surfaces passes through the cornea and the aqueous humor, reaching the crystalline lens. As such, a third Purkinje image (PIII) (larger than the previous Purkinje images) is formed by the reflection of light from the anterior surface of the lens. Finally, a fourth Purkinje image (PIV) is formed by light reflected on the anterior surface of the crystalline lens. This PIV image is similar in size to PI. However, due to the air–cornea interface of the last surface of the crystalline lens, the difference in the refractive index causes the luminous intensity of PIV to be less than that of PI [31,32].

Purkinje images are generally used to measure eye behavior [33,34,35]. This is achieved by capturing the reflections generated with light that is projected on the eye. In this research, Purkinje images are used to generate two patterns on the eye (each one at different wavelength in the visible spectrum). Subsequently, the differences generated by both patterns are calculated through the Hartmann test. If the direction of the light is related to the refractive index, it would provide a guideline for how the light of both patterns varies in the last surface of the crystalline lens, allowing measurement of the chromatic aberration.

By comparison, in the case in which the refracted light in the Purkinje IV reaches the retina, this is reflected in the last surface of the crystalline lens. According to the article presented by P. Artal et al., when a double-pass system is used in color experiments, a phenomenon called chromatic-aberration cancellation occurs [36,37]. This phenomenon causes the TCA to be canceled by the second beam light that passes through the eye’s optical system. In our system, the light that is refracted towards the retina obtains the complete information of the chromatic aberration produced in the eye. However, the reflection generated from the retina towards the last surface of the lens produces a new measurement of the aberration that is canceled by the aberration generated previously. Thus, we finally only have the chromatic aberration generated from the first surface of the cornea towards the last surface of the crystalline lens.

### 2.2. Hartmann’s Test

**Figure 2:**
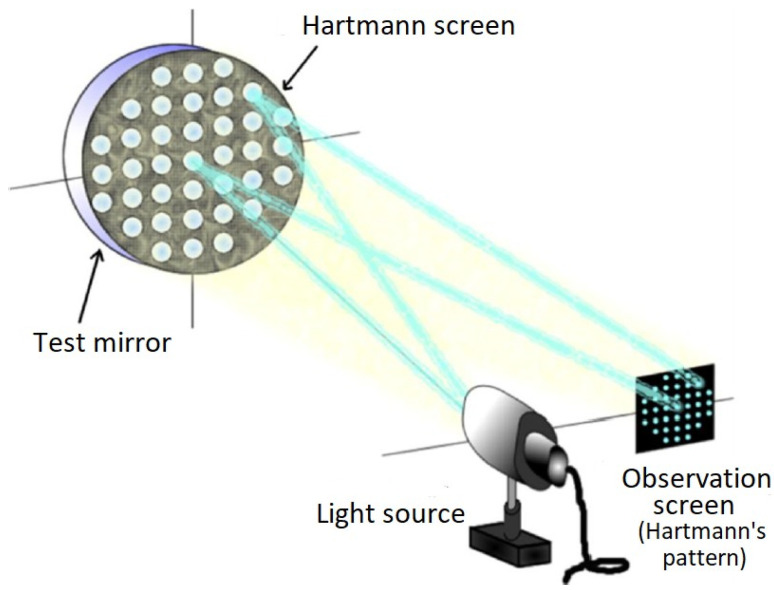
Hartmann test applied to optical elements.

Currently, Hartmann’s test is used to measure the wavefront or shape of an optical surface [38]. The basic setup of the Hartmann test is shown in Figure 2. The aspherical wavefront diverges from the light source and heads towards the test mirror, but before reaching the mirror it encounters the Hartmann screen, which lets only a few light beams pass (one for each hole on the screen). The rays that manage to pass are reflected into the mirror and sent back through the same holes of the Hartmann screen. These rays finally arrive at the observation screen, which can be an opaque screen, a photographic film, or a Charge-Couple Device (CCD) sensor of a video camera, where the Hartmann pattern (which is a dot diagram) is recorded. Once the Hartmann pattern is obtained, the next step is to measure the wavefront or the wavefront aberration. This can be achieved by measuring the point positions of the Hartmann pattern generated from a test mirror. These positions are then compared with the point positions of the Hartmann pattern generated by a reference mirror.

**Figure 3:**
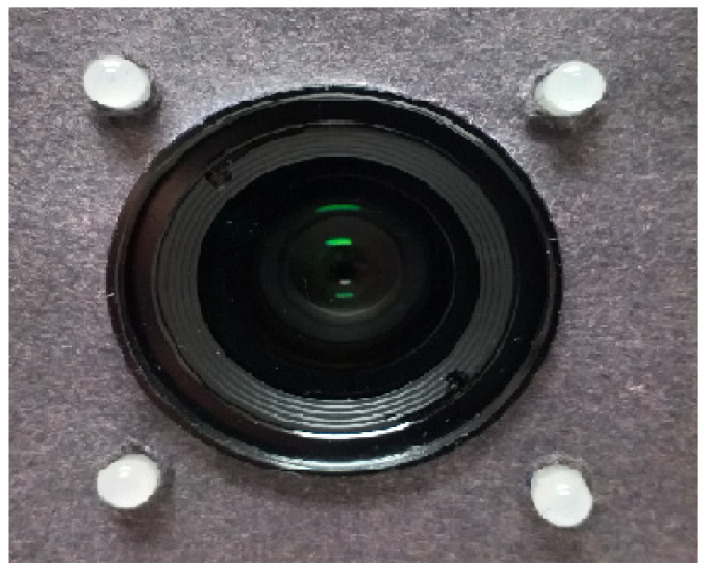
Hartmann screen.

For this research, the Hartmann screen contains four holes, where the observation screen is a complementary metal-oxide-semiconductor (C-MOS) camera, and the light source is generated by four LEDs (placed forming a square) arranged on the Hartmann screen, as seen in Figure 3. In this study, the Hartmann test is used to measure the difference generated by both patterns (by comparing each point of the pattern generated with the blue pulse against each of the points of the pattern generated with the red pulse), and the Zernike polynomials $Z_{i}\left(x,y\right)$ are used to obtain the wavefront W(x,y) and calculate the LCA.

In Table 1, the LCA can be measured using the Zernike coefficients. According to Thibos et al. [1], the LCA is defined as the difference in the coefficient of defocus ($C_{4}$ coefficient) at different wavelengths.

## 3. Description of the Measurement System

**Figure 4:**
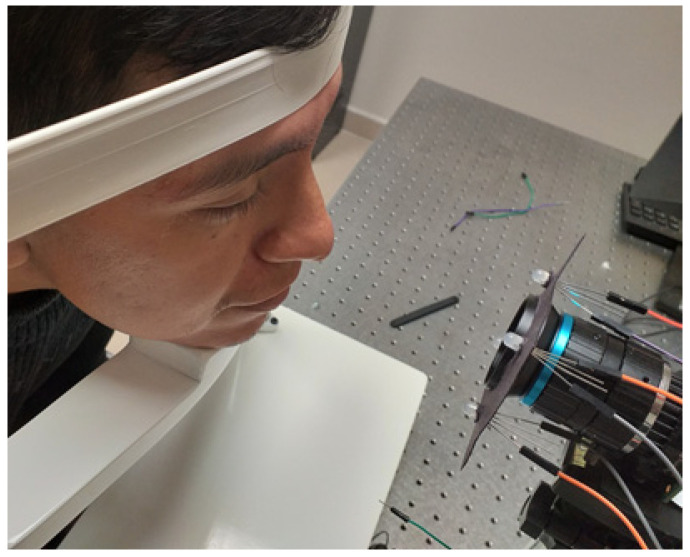
Subject under test.

A modified prototype was built based on the device described in the article submitted by Jean-Cyriaque Barry et al. [39]. A quadrangular array of LEDs was used instead of three-lined sources since a linear array would not provide enough information to describe the wavefront (the LEDs had a 5 mm diameter, with a pulse width of 440–480 nm and 580–640 nm, a 12° viewing angle, and maximum output power of 25 mW to avoid human eye damage). As mentioned, the quadrangular array of LEDs was designed to illuminate the test subject’s right eye, aligned with a varifocal lens and a C-MOS camera (12.3-megapixel SONY IMX477 sensor, 1.55 μm×1.55 μm pixel size), which captures the Purkinje images produced by the light source. The use of a quadrangular arrangement has some advantages over the linear array used in the work on which this investigation is based. For instance, it has a symmetrical shape, which easily allows the identification of the Purkinje images (PIV is inverted concerning the PI image). Furthermore, the geometric pattern can be used to reconstruct the wavefront of the system being analyzed. The constructed instrument was mounted on a base that carries a chin and forehead rest designed to hold the subject’s head steady during measurements; Figure 4. In this setup, the PII is not visible because it is very close to the PI and the PI covers the PII. Furthermore, the PIII is not visible since the image is subjected to pre-processing that highlights the edges (especially those of the pupil) and noise reduction that removes the PIII from the eye.

**Figure 5:**
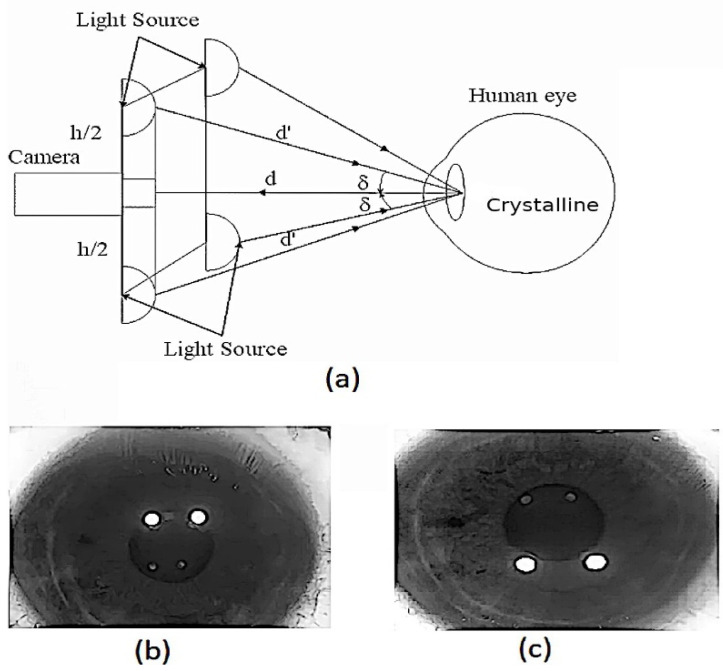
Schematic representation of a prototype setup for Purkinje IV reflection pattern evaluation with light sources pointing at the eyes from above. (**a**) The light sources are separated by the distance, h and $\frac{h}{2}$, from the camera. In this setup, d and $d^{\prime }$ form an angle δ. that allows us to see the Purkinje images (PI and PIV). (**b**) Results obtained when used to impinge on the light source placed on top. (**c**) Results obtained when used to impinge on the light source placed below.

**Figure 6:**
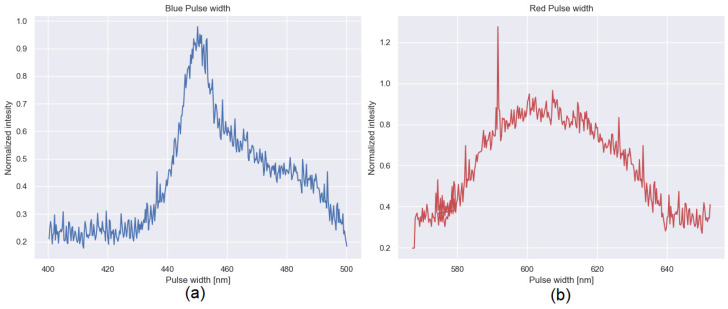
Normalized spectra of LEDs with different pulse widths: (**a**) Blue light source (440–480 nm) and (**b**) red light source (580–640 nm).

The measurement procedure begins by aligning the test subject with the previously described device. The subject is aligned in such a way that the pupil is centered within the recording area of the C-MOS camera Figure 5a. This aligns the line of sight with the axis of the instrument. Following this, a first test is conducted in which the LEDs placed above the camera are turned on to perform the first acquisition of the PIV seen in the lower part of the pupil, as seen in Figure 5b. Finally, the LEDs above the camera are turned off and the LEDs placed below the camera are turned on to generate the PIV; however, at this time (since the shape of the screen is symmetrical, the LEDs cannot be turned on at the same time, as it would cause both PI and PIV to overlap), they can be seen in the upper part of the pupil Figure 5c. This procedure is carried out for two different pulse widths (blue 440–480 nm and red 580–640 nm), as shown in Figure 6. Then, each pattern that we generated through the pupil is used to measure the difference between each of them.

### Test Subjects

Five subjects (S1: female; S2: female; S3: male; S4: male; and S5: male) participated in this study. The test subjects did not have trauma or eye surgery. All measurements were made on the right eye.

All test subjects were informed about the test objective and proper consent was obtained. The experiment procedures were developed according to tenets of the Declaration of Helsinki. The power generated by the quadrangular array of LEDs was less than 25 mw. This power is below the maximum allowed according to the American National Standards Institute [40].

## 4. Purkinje Image Detection

**Figure 7:**
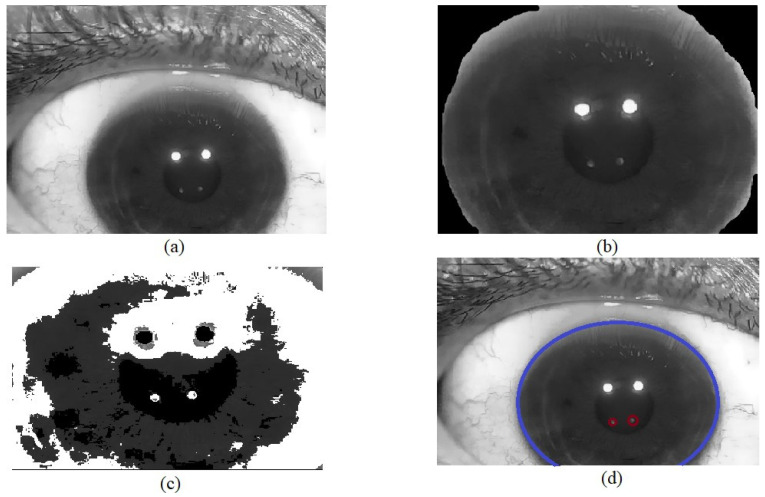
Segmentation process once the PIV image of Purkinje is formed: (**a**) image obtained from the wave-front sensor; (**b**) iris zone segmentation; (**c**) applying a threshold and noise removal algorithm to eliminate unwanted areas in the image; (**d**) detection of PIV in the pupil area.

**Figure 8:**
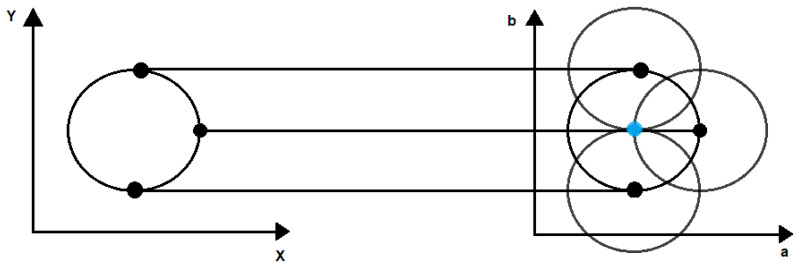
The Hough transform principle.

Purkinje images are visible on the pupil as shown in Figure 7a. However, the Hough transform was applied to extract the section of the image that corresponds to the iris of the eye and eliminate the rest of the image content obtained by the camera [41]. This prevents the detection of false reflections generated by other parts of the human eye (tear film). The Hough transform was selected since it is an algorithm used for feature detection (including lines and circles) in a digitized image [42]. According to the Hough transformation for circles, each pixel in the image corresponds to a circle in Hough space. All the points of circle C in the image are transformed into several circles with the same radius r, where their intersection, O, is the center of the detected circle, as represented in Figure 8. The Hough transform results are stored in a matrix known as the Hough accumulator. The accumulator values are updated for each circle generated using the Hough transform. The center O of the detected circle C is the maximum value generated in the accumulator circle (where circle C represents the area of the iris). Once the iris area is extracted (Figure 7b), the next step is to find PIV. This is required to establish the thresholds that allow PIV to be highlighted and eliminate PI. In the image of the eye (Figure 7c), PIV has an intensity ranging between 0 and 100 pixels, whereas PI has an intensity ranging between 200–255 (in grayscale); these thresholds highlight PIV. Finally, applying the Hough transform again, but now to find a smaller radius, we can obtain the form of the PIV (as represented in Figure 7d). Finally, to obtain the center point within the PIV, we can calculate the centroids by adding all the positions of the pixels and dividing them by the total number of pixels found by the Hough transform. These centroids are used to calculate the difference generated by each pattern and, subsequently, to calculate the LCA using the Zernike polynomials.

## 5. Wavefront Measurement Using a Modal Method with Zernike Polynomials and Local Slopes

The coordinates obtained from PIV (the centroids coordinates) were used by the system and a modal estimation was carried out to calculate the LCA. Thus, to measure the longitudinal chromatic aberration, the difference generated by both patterns (the pattern generated by a pulse width of 440–480 and the pattern generated by a pulse width of 580–640) must be calculated. The local slopes, or partial derivatives, of the wavefront, can be measured by shifting the focal points (in this case the centroids located in the PIV for both pulse widths). The partial derivatives of the wavefront W(x,y) in the positions (x,y) of the image are obtained from by Equations (1) and (2):

(1) $$TA_{x}=\frac{\partial W\left(x,y\right)}{\partial x}$$

(2) $$TA_{y}=\frac{\partial W\left(x,y\right)}{\partial y}$$

where $TA_{x}\left(x,y\right)$ and $TA_{y}\left(x,y\right)$ are the evaluated values of the slopes. Many approximations have been proposed in the literature for wavefront reconstruction using partial derivatives. In this case, Zernike polynomials are used as a method to reconstruct the wavefront [43,44]. The applied wavefront W(x,y) according to Liang et al. [43] is expressed as (3):

(3) $$W\left(x,y\right)=\sum_{i=0}^{5}C_{i}Z_{i}\left(x,y\right)$$

where $Z_{i}\left(x,y\right)$ is the Zernike polynomial, up to degree five, and $C_{i}$ are the coefficients of the Zernike modes [45]. From (3), the partial derivatives can be expressed as Equations (4) and (5):

(4) $$TA_{x}=\frac{\partial W\left(x,y\right)}{\partial x}=\sum_{i=0}^{5}\frac{C_{i}\partial Z_{i}\left(x,y\right)}{\partial x}$$

(5) $$TA_{y}=\frac{\partial W\left(x,y\right)}{\partial y}=\sum_{i=0}^{5}\frac{C_{i}\partial Z_{i}\left(x,y\right)}{\partial y}$$

Using the method of least squares on Equation (3) with the derivatives provided in Equations (4) and (5), the coefficient values are obtained according to Equation (6) through Equation (10):

(6) $$C_{1}=\frac{1}{2}\;\left[TA_{xa}+TA_{xb}+TA_{xc}+TA_{xd}\right]$$

(7) $$C_{2}=\frac{1}{2}\;\left[TA_{ya}+TA_{yb}+TA_{yc}+TA_{yd}\right]$$

(8) $$C_{3}=\frac{1}{4\sqrt{2\rho }}\;\left[-TA_{xa}+TA_{xb}-TA_{xc}+TA_{xd}\right]$$

(9) $$C_{4}=\frac{1}{4\sqrt{2}\rho }\;\left[TA_{xa}-TA_{xc}+TA_{xb}-TA_{xd}-TA_{ya}+TA_{yb}-TA_{yc}+TA_{yd}\right]$$

(10) $$C_{5}=\frac{1}{4\sqrt{2}\rho }\;\left[TA_{ya}-TA_{yc\;}+TA_{yb}-TA_{yd}\right]$$

In Equations (6)–(10), $x_{a}-x_{d}$ and $y_{a}-y_{d}$ represent the slope measurements at each of the points in the quadrangular array. ρ is the distance from the origin to each of the four points in the quadrangular array. The Zernike coefficients, the wavefront distribution, and the contribution of the individual Zernike modes can be calculated with the known derivatives.

### Chromatic Aberration Measurement

The crystalline lens pattern information for both pulse widths for the C-MOS device was collected. The system processes the information and transforms the pattern information into Zernike coefficients. According to Yangchung et al. [46], LCA can be calculated using Equation (11):

(11) $$D=D_{b}-D_{r}=\frac{4\sqrt{3}\left(C_{b4}-C_{r4}\right)}{R^{2}}$$

where D is the LCA (in diopter), $D_{b}$ is the blue light’s defocus, $D_{r}$ is the red light’s defocus, $C_{b4}$ is the blue light’s coefficient defocus (in μm), $C_{r4}$ is the red light’s coefficient defocus (in μm) and R is the pupil radius.

## 6. Results

LCA results of the five different eyes in the central field of view are shown in Table 2. The average LCA was 0.4984 D.

To measure the viability of the device, the repeatability error was used. Different measurements at the same eye position were used to measure the repeatability error. This error is expressed as the standard deviation. The measurement consists of performing the same test on different days and measuring the difference that exists between the patterns of points generated in the eye. The standard deviation of blue pulse width was 0.102 mm, whereas that of the red pulse width was 0.16 mm.

### 6.1. Discussion

Table 3 shows LCA data reported from different tests. These studies use different conditions (different spectral ranges), which complicates the comparison of the measurements obtained in this study.

In addition, the LCA obtained in this study can be compared with subjective techniques. According to Atchison et al. [28], the longitudinal chromatic dispersion equation can provide a reference value that can be compared with our objective method. The equation is described by Equation (12):

(12) $$R_{x}\left(\lambda \right)=1.60911-\frac{6.70941x10^{5}}{\lambda^{2}}+\frac{5.55334x10^{10}}{\lambda^{4}}-\frac{5.59998x10^{15}}{\lambda^{6}}$$

where $R_{x}\left(\lambda \right)$ is the chromatic difference of refraction. In this study, using the chromatic difference equation, LCA, calculated between 440 and 640 nm, was 0.3953 D (compared with the LCA measurement value of 0.4984 D). The equation presented by Atchison et al. [3] was based on subjective methods derived from the equation of Thibos et al. [14]. Nevertheless, the results of this research were obtained by an objective method. The LCA results obtained in this study were higher than those obtained using the longitudinal chromatic dispersion equation. This result cannot be due to the fact the subject is not immobile during the test, which would generate a small error when the information is processed. Furthermore, the measurements made in this work were only obtained up to the last surface of the crystalline lens, so we could say that monochromatic aberration is more present before reaching the last part of the eye (humor vitreous retina).

### 6.2. Implication of Longitudinal Chromatic Aberration in the Generation of New Devices

The techniques used to measure LCA have made it possible to improve the quality of vision in the human eye. Studies presented in this work showed the importance of measuring longitudinal chromatic aberration in the visible spectrum. This importance lies in the design of new devices that allow diagnosing and improving the quality of vision in the human eye (new wavefront sensors and intra-ocular lenses). Developing new techniques to measure longitudinal chromatic aberration provides a new possibility to develop new devices that allow the quality of vision to be measured accurately and at a lower cost.

### 6.3. Spot Detection

**Figure 9:**
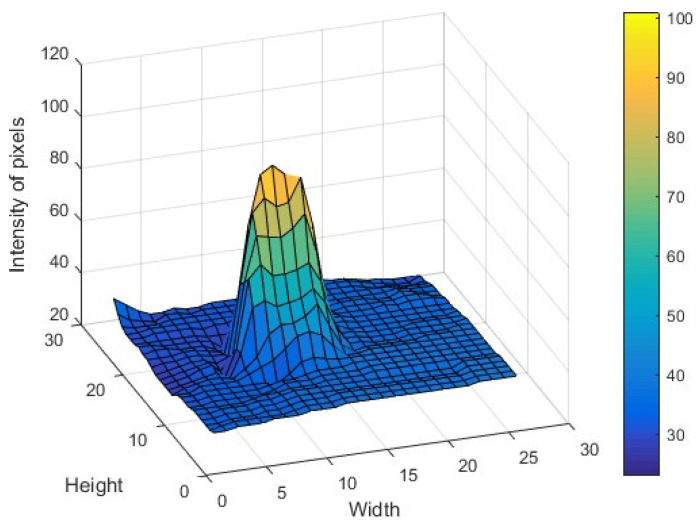
Intensity distribution of the focus spots in an image pattern for the tested eye. Width and height are in pixels.

Figure 9 shows the intensity of the spot on the last crystalline lens layer. To determine the position of the spot, we use Hough’s algorithm, which is widely used in the detection of shapes such as the iris and the pupil [48,49]. The light distribution of the spot depends on the wavefront within the lens aperture. Since the wavefront function does not change within the aperture lenslet, it can be expressed in a few terms of the Zernike polynomial, which consist of the local inclinations, the defocus, and the astigmatisms values. The local tilt terms vary proportionally according to the focal point of the camera lens, while the defocus term will change the light distribution. However, the central point of light distribution will not vary. By comparison, the camera lens is small enough that high order aberrations can be ignored, and the centers found in the light distribution will provide an adequate image for estimating the position of the spot. One of the advantages of the Hough transform is its ability to locate the centroids in different intensities, which allows greater accuracy when measuring slopes. Such accuracy is difficult to achieve by any other method.

### 6.4. Degree in the Zernike Polynomials

According to the article presented by Malacara et al., a four-point array provides enough information to detect fundamental frequency components (low-order aberrations) [50]. However, limited information is available to detect second harmonic components (high order aberrations). These components can be obtained from a four-point arrangement, if additional information, such as the phase between harmonic components, is known. Unfortunately, this is not possible with the current test.

## 7. Conclusions

A system for an objective measurement of longitudinal chromatic aberrations of the human eye was presented. The system uses a pattern of light (at different pulse widths) generated on the last surface of the lens. This pattern of points has a quadrangular shape and is compared with a reference pattern to measure the difference between each of them. Subsequently, these displacements (slopes) are used in conjunction with the Zernike polynomials to reconstruct the wavefront. Once the wavefront is reconstructed, the Zernike coefficients are used (specifically, coefficient 4) to obtain the longitudinal chromatic aberration (LCA). The data obtained from the LCA can help to improve the design of the intra-ocular lens. In addition, the longitudinal chromatic aberration can be used in fundus photography; by compensating for longitudinal chromatic aberration, the quality of the images obtained is improved. Moreover, the technique described in this work can be used to measure chromatic aberration in lenses.

## Figures and Tables

**Table 1:** Zernike coefficients in Cartesian coordinates, $C_{0}-C_{5}$.

Term	Zernike Polynomial	Meaning
$C_{0}$	1	Constant term
$C_{1}$	x	Tilt in *x*-direction
$C_{2}$	y	Tilt in *y*-direction
$C_{3}$	2xy	Oblique primary astigmatism
$C_{4}$	$-1+2x^{2}+2y^{2}$	Defocus
$C_{5}$	$-x^{2}+y^{2}$	Vertical/Hor. Primary astigmatism

**Table 2:** Longitudinal chromatic aberrations between blue pulse width (440–480 nm) and red pulse width (580–640 nm) lights.

Subject	Age	LCA(D)
S1	24	0.4953
S2	26	0.4949
S3	24	0.5013
S4	28	0.5044
S5	30	0.4963
Average	26.4	0.4948

**Table 3:** Comparison of the results obtained with different techniques established in the literature (S: subjective method, O: objective method).

Study	Number of Subjects	Wavelength (nm)	Average LCA (D)
Manzanera et al. [47] (O)	3	440–694	1.75
Vinas et al. [13] (O)	5	488–700	1.00
Vinas et al. [13] (S)	5	488–700	1.51
Fernández et al. [29] (O)	5	700–950	0.45
This study (O)	5	440–640	0.49

## Data Availability

The data are open and will be given upon reasonable demand.
